# Multiple‐Level Variation of Advertisement Calls of *Microhyla fissipes* Across Hainan Island

**DOI:** 10.1002/ece3.71342

**Published:** 2025-05-08

**Authors:** Yujuan Guo, Tianyu Qian, Yulong Li, Keji Guo, Wenbo Zhu, Bin Wang, Jianping Jiang

**Affiliations:** ^1^ Chengdu Institute of Biology Chinese Academy of Sciences Chengdu China; ^2^ University of Chinese Academy of Sciences Beijing China; ^3^ Central South Inventory and Planning Institute of National Forestry and Grassland Administration Changsha China

**Keywords:** anuran communication, bioacoustics, call variation, frogs

## Abstract

Vocalization is an important feature in anuran identification that could vary among individuals and populations. We present an investigation of multiple‐level variation on advertisement calls of *Microhyla fissipes* from nine populations across Hainan Island and further test the differences between the two geographical groups that were divided by morphological features in a previous study. We found that dominant frequency is the most static call parameter at the individual level. Four of six call parameters show significant differences between groups and could be useful for identification between groups. The southwest (SW) group from Hainan Island represents the highest dominant frequency among all reported advertisement calls of *M. fissipes* in the literature, implying a need for further studies.

## Graphical abstract

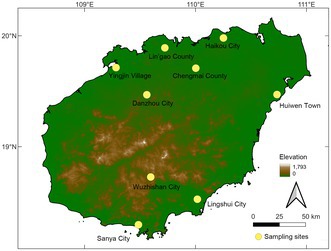

## 1 Introduction

In most anurans, acoustic signals are important in transmitting information about breeding, territorial, or other social contexts from the emitter to the receiver (Wells 2007). The most frequently documented acoustic signal in frogs is the male advertisement call that is used to attract female frogs for breeding attempts (Köhler et al. 2017). Such signals play an important role in species identity, contributing to premechanism in breeding isolation. Thus, variation in advertisement calls from different populations could be regarded as a sign of taxonomic uncertainty. For several decades, studies on variation in advertisement calls among geographical populations have revealed taxonomic changes, environmental adaptations, and provided evolutionary evidence (Littlejohn 2008; Littlejohn and Loftus‐Hills 1968; Röhr et al. 2020; Smith and Hunter 2005).

*Microhyla fissipes* Boulenger (1884) is a small frog species (average SVL 22 mm in males, and 23 mm in females) (Fei et al. 2009). However, it has successfully expanded its distribution range throughout eastern China to northern Vietnam (Frost 2024). The accelerated development ability may have contributed to its large expansion in warm areas—eggs of *M. fissipes* were reported to hatch within 24 h (under 25°C–28°C), and the metamorphosis could happen within 20–30 days (Fei et al. 2009). In their breeding season, male *M. fissipes* emit loud calls forming choruses near temporal water bodies, often hiding under vegetation roots or soil burrows (Fei et al. 2009).

The hilly topography of Hainan Island has made geographical barriers among populations of *M. fissipes* . Li et al. (2017) found that *M. fissipes* from Hainan Island could be classified into two morphological groups—the northeast (NE) group with a larger body size and the smaller southwest (SW) group. Further study by Chen et al. (2025) has revealed karyotype differences between the two groups, which they implied are derived from the temperature adaptations. However, temperature and body size are both crucial impactors in shaping the phenotypes of anurans' calls, but no reliable analysis on advertisement calls across Hainan Island is currently available.

Therefore, in this study, based on advertisement calls collected from nine localities across Hainan Island, we first tested call variation across multiple levels, including individual, population, geographical, and overall Hainan Island levels. Second, we tested for differences in call parameters between the two geographical (and morphological) groups.

## 2 Materials and Methods

### 2.1 Call Recording and Processing

**FIGURE 1:**
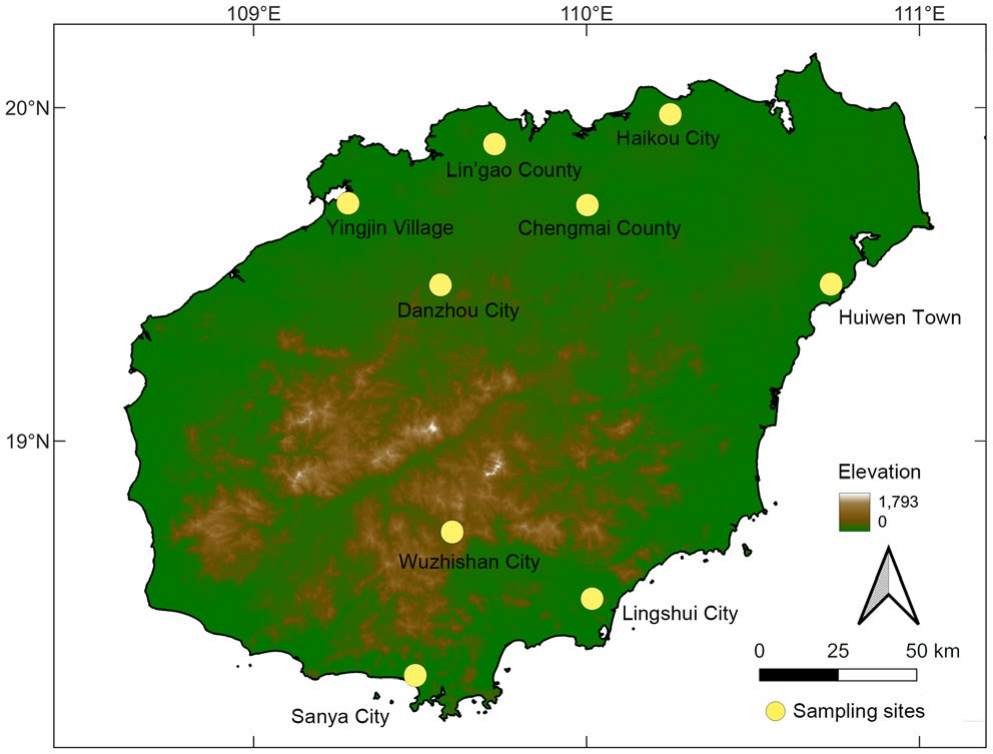
Map of Hainan Island showing sample sites for this study.

Field recordings were made from May to Aug 2016 from nine localities on Hainan Island (Figure 1). While the male frogs were calling between 20:00 and 24:00 h, we placed a Marantz PMD 661 digital recorder coupled with a Sennheiser ME66/K6 shotgun microphone directed to a calling male frog at a distance of 10–15 cm. After placing the recorder and microphone, the researchers moved at least 0.2 m from the microphone for 5–10 min to minimize anthropogenic disturbance during the recording. The calling individual was not collected; thus, we did not record the data on its body condition. After removing the recordings with overlapped individual calls, calls from a total of 33 individuals were counted. Sampling localities, coordinates, number of males recorded, air temperature, and recording date are shown in Table 1.

**TABLE 1:** Sampling information for advertisement calls of *Microhyla fissipes* across Hainan Island.

Group	Locality	Longitude	Latitude	Air temperature (°C)	Number of recorded males	Recording date
NE	Haikou City	110.25163	19.98020	27.9	3	2016/5/28
	Huiwen Town	110.73444	19.47056	25.8	2	2016/8/11
	Chengmai County	110.00269	19.70805	27.0	4	2016/5/29
	Lin'gao County	109.72374	19.89148	26.4	3	2016/5/30
	Danzhou City	109.56134	19.46854	27.0	4	2016/6/01
	Yingjin Village	109.28362	19.71347	28.9	5	2016/5/31
SW	Wuzhishan City	109.59653	18.72689	26.7	4	2016/8/05
	Lingshui City	110.01676	18.52668	28.7	6	2016/8/08
	Sanya City	109.48570	18.29662	29.0	2	2016/6/09

The collected recordings were processed in Adobe Audition 3.0 to reduce noises from low‐band frequencies. The filtered WAV files were then processed in Raven Pro 1.6.5 with a “Default 1.3 power” window preset, which means a “Hann” window with 512 samples, an overlap of 50%, and a Hop size of 256 samples. The terms and definitions of acoustic analysis follow the “call‐centered” definition by Köhler et al. (2017). We measured six call parameters, including call duration, call interval, dominant frequency, bandwidth between 5% and 95%, pulse number within a call, and pulse rate (pulse/s) computed by dividing (“pulse number”‐1) by the duration between the onset of the first pulse and the onset of the last pulse of the call (following Bee et al. 2012). For each male, we selected five continuous, high signal‐to‐noise ratio calls for measuring the above parameters.

### 2.2 Data Analysis

We used the coefficients of variation (CV = 100% * [SD/X]; SD is the standard deviation of the distribution, and X is the mean of the distribution) to score the variation of call parameters on different levels following Kaefer and Lima (2012). First, the individual‐level variation (CV_i_) was calculated by dividing the SD of each call parameter for each frog individual by their average value (X). Then we calculated the population‐level variation (CV_p_) and group‐level variation (CV_g_) based on the average values and SDs of overall populations and groups. Finally, we calculated the overall‐level variation (CV_o_) from the grand mean and SD based on all individual means from our samples. We determined the ratio between the overall and group variation as CV_o_/CV_g_. The CV was categorized as “dynamic” (CV_i_ > 12%), “intermediate” (5% ≤ CV_i_ ≤ 12%), and “static” (CV_i_ < 5%) following Gerhardt (1991). We performed model II ANOVAs to provide additional estimates of which call parameters vary overall level than group levels (Beecher 1989; Prasad et al. 2022). The Model II ANOVAs were generated in R by using the *Anova* function of *car* package v.3.1.1 (Fox et al. 2019). Statistical values were obtained through *anova_stats* function of *sjstats* package v. 0.19.0 (Lüdecke 2024). We then generated boxplots of multiple level variation on each call parameter by using *ggplot* function in *ggplot2* package v. 3.4.1 (Wickham 2016) through R.

Temperature has been reported to affect call performance in most anurans (Wells 2007). Prior to comparing the differences between two geographical (and morphological) groups (NE and SW), we calculated overall regression coefficients (b) for all call parameters by using the *lm* function in R. For variables that exhibit significant relationships with the temperature, a temperature‐corrected value is used for the comparison between the two groups. We used the equation proposed by Platz and Forester (1988) and calculated the temperature‐corrected value at a mean temperature of 27.6°C. Finally, each call parameter from our dataset was temperature‐corrected except for the pulse number.

To test the correlation between acoustic parameters and geographic distance between different populations of *M. fissipes* , we applied a Mantel test (Mantel 1967) for each acoustic parameter, which was performed by using the *vegan* (Oksanen et al. 2022) and the *geosphere* (Hijmans 2023) package. In the correlation matrix, we included geographical coordinates of each locality and the mean acoustic parameters for each population.

To compare the differences in each call parameter between the two groups, we used a T‐test or Mann–Whitney's U test (depending on variable distribution) and applied the *t.test* function or *wilcox. test* function in R to verify differences in each call. The distribution of each variable was estimated through Shapiro–Wilk's test by using the *shapiro.test* function in R. For variables with normal distribution, Bartlett's test (with the *bartlett. test* function in R) was used to test the homogeneity of variance. For variables with non‐normal distribution, we used Levene's test (by using *leveneTest* function in *car* package). All variables agreed with the homogeneity test of variance (*p* > 0.05). Additionally, Kernel density estimate plots were used to display the distribution of each call parameter between the two groups, which were performed by using the *ggplot* function. Additional enhancements on each plot were applied by using the *ggpubr* (Kassambara 2023) and *ggthemer* (Tobin 2024) packages.

## 3 Results

### 3.1 Variation in Advertisement Calls

**FIGURE 2:**
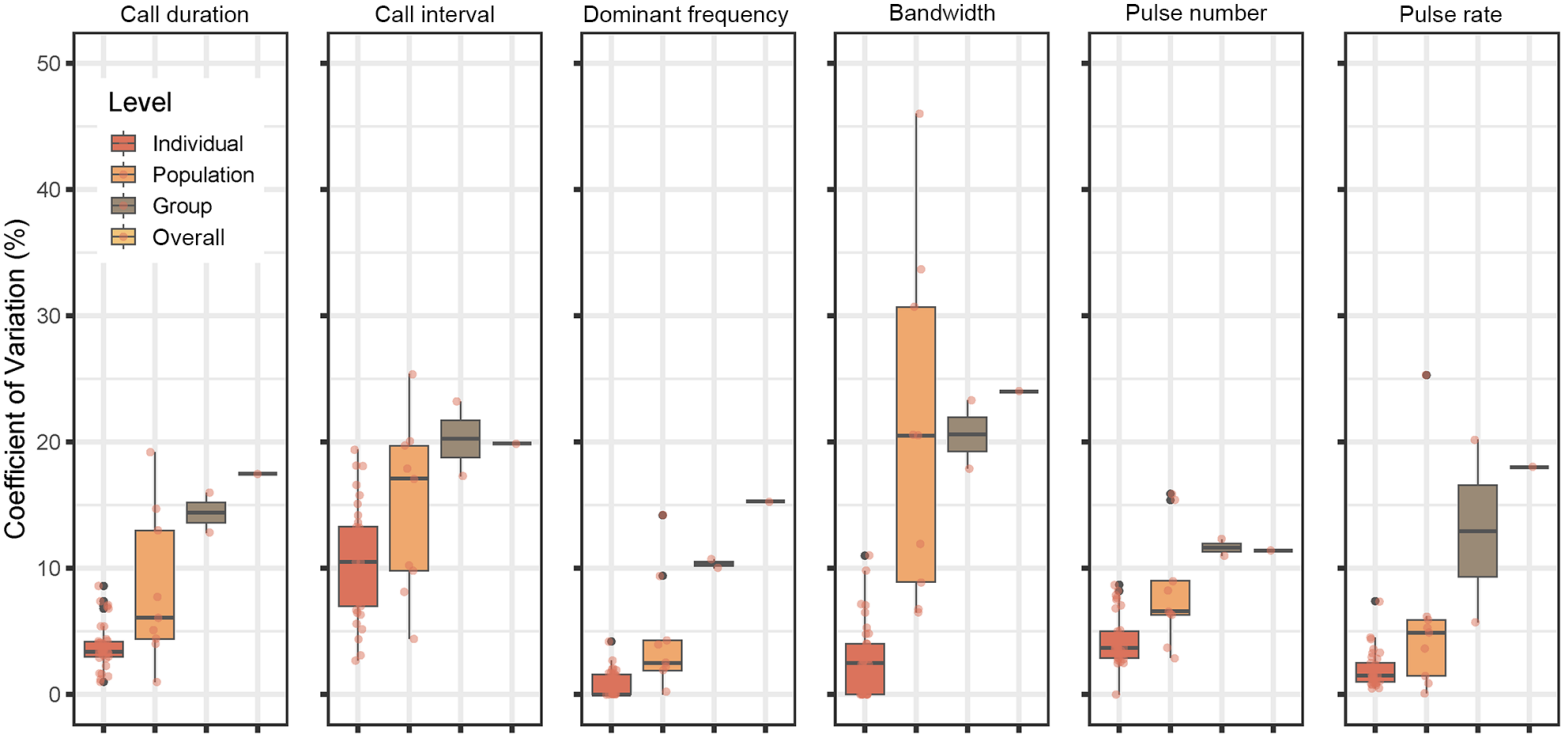
Multiple‐level coefficient of variation on call parameters of *Microhyla fissipes* .

Detailed descriptive statistics on advertisement calls of *M. fissipes* are shown in Table 2. Five of six call parameters with CV_i_ < 5% were categorized as static in *M. fissipes* , while the call interval stands as an intermediate parameter with an average CV_i_ of 10.4. Figure 2 shows the differences of variation between each level on six call parameters. The population‐level variation of bandwidth has a larger value than the group level, indicating the variation within the populations was larger than that within the groups. The CV_o_/CV_g_ value provides an estimate of the possibility of identification between groups, which shows that call duration (4.49), dominant frequency (19.13), bandwidth (8.57), and pulse rate (9.00) are effective parameters to determine whether an individual belongs to a particular group. The Model II ANOVA shows similar results that dominant frequency was the most effective parameter in group identification (*p* < 0.001, *η* ^2^ = 0.55), followed by pulse rate (*η* ^2^ = 0.280), bandwidth (*η* ^2^ = 0.256), and call duration (*η* ^2^ = 0.257) which all show significant differences (*p* < 0.05). The results of the Mantel test indicated that none of the six acoustic parameters showed a significant correlation with geographic distance between populations of *M. fissipes* (Table 3).

**TABLE 2:** Descriptive statistics on advertisement calls of *Microhyla fissipes* from Hainan Island.

Call parameter	Mean	SD	Range	CV_i_ (%)	CV_p_ (%)	CV_g_ (%)	CV_o_ (%)	CV_o_/CV_g_	*p*	*η* ^2^
Call duration (ms)	215.1	37.6	147.3–291.4	3.9 (1.0–8.6)	8.3 (1.0–19.2)	12.6 (11.5–13.6)	17.7	4.49	**0.003**	0.257
Call interval (ms)	272.7	54.3	189.5–409.3	10.4 (2.7–19.4)	14.7 (4.4–25.4)	21.5 (18.1–24.9)	22.5	1.91	0.277	0.038
Dominant frequency (Hz)	3416	522	2550–4313	0.8 (0.0–4.2)	4.3 (0.0–14.2)	8.9 (8.3–9.6)	15.1	19.13	**< 0.001**	0.550
Bandwidth (Hz)	2059	494	1050–3000	2.8 (0.0–11.0)	20.6 (6.5–46.0)	20.2 (15.6–24.9)	23.9	8.57	**0.003**	0.256
Pulse number	14.0	1.6	11.2–17.6	4.1 (0.0–8.7)	8.3 (2.9–15.9)	11.8 (11.2–12.3)	12.1	2.78	0.467	0.017
Pulse rate (pulses/s)	64.1	11.5	39.2–79.9	2.0 (0.5–7.4)	6.0 (0.1–25.3)	12.4 (5.7–19.1)	17.9	9.00	**0.002**	0.280

*p* values in bold indicate significant differences between groups.

**TABLE 3:** Results of Mantel test evaluating which acoustic parameters of the advertisement call of *Microhyla fissipes* are correlated to the geographic distance between populations (r: A positive value indicates that larger geographic distance corresponds to greater differences in acoustic parameters).

Source	*r*	*p*
Call duration (ms)	−0.256	0.9515
Call interval (ms)	0.006	0.4734
Dominant frequency (Hz)	−0.249	0.9617
Bandwidth (Hz)	−0.181	0.8286
Pulse number	−0.348	0.9926
Pulse rate (pulses/s)	−0.164	0.8103

### 3.2 Call Differences Between NE and SW Groups

**FIGURE 3:**
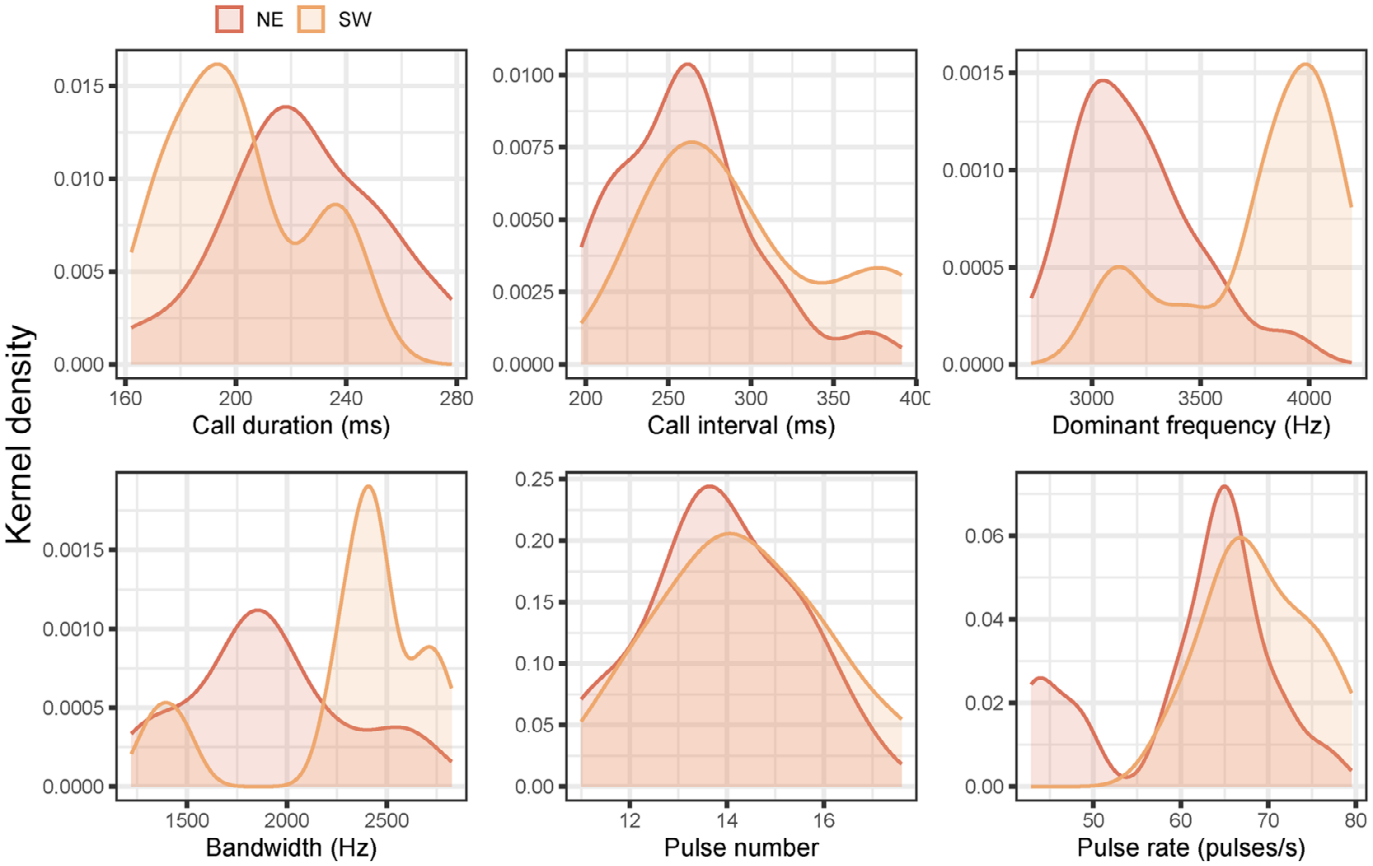
Plots of kernel density estimate showing the differences of advertisement calls between two groups (NE and SW).

By using temperature‐corrected values, four of six call parameters showed significant differences between NE and SW groups (*p* < 0.05) (Table 4). Compared to the SW group, the NE group has a longer call duration (mean value 224.3 ms vs. 201.0 ms), lower dominant frequency (mean value 3190 Hz vs. 3783 Hz), narrower bandwidth (mean value 1908 Hz vs. 2307 Hz), and lower pulse rate (mean value 60.9 pulses per second vs. 69.1 pulses per second). The Kernel density estimate plots (Figure 3) show a large overlap in call interval and pulse number, with distinct differences in dominant frequency and bandwidth.

**TABLE 4:** *T*‐test/Wilcoxon‐test results compare each call parameter in two groups (NE and SW) from Hainan Island.

Call parameter	Mean value (NE vs. SW)	Method	*p*
Call duration (ms)	224.3 vs. 201.0	*T*‐test	**0.020**
Call interval (ms)	260.4 vs. 296.3	Wilcoxon‐test	0.096
Dominant frequency (Hz)	3190 vs. 3783	Wilcoxon‐test	**< 0.001**
Bandwidth (Hz)	1908 vs. 2307	Wilcoxon‐test	**0.019**
Pulse number	13.9 vs. 14.3	*T*‐test	0.485
Pulse rate	60.9 vs. 69.1	Wilcoxon‐test	**0.016**

*p* values in bold indicate significant differences between groups.

## 4 Discussion

In this study, we present a first evaluation on multiple‐level variation of advertisement calls of *M. fissipes* across Hainan Island. Our results suggest a relatively low variation of most call parameters at the individual level. However, the observed individual variation suggests that the advertisement call could be individually specific. Especially when comparing the values between two geographical groups, the significant differences in spectral parameters (dominant frequency and bandwidth) could be partially explained by geographical variation. The largest value of CV_o_/CVg was present at dominant frequency, which could be the most useful parameter for identification among the two groups.

Previous studies have shown that the NE group has a larger body size (Chen et al. 2025; Li et al. 2017). Thus, the lower dominant frequency and narrower bandwidth produced by the NE population could be due to the larger body size, since larger individuals produce calls of lower frequencies due to physical limitations (Walkowiak 2007). Chen et al. (2025) have thoroughly investigated the genetic differences between the NE and SW groups, resulting in different karyotypes that could be divided into tetraploid (NE) and diploid (SW) forms. They also performed acoustic analysis among genetically identified individuals but did not provide any sampling information. According to their spectrograms (Chen et al. 2025: figure S4), the tetraploid individuals performed lower call frequencies, which may be due to the effect of body size as well.

Currently, studies on advertisement calls of *M. fissipes* have mostly focused on the population from eastern China (Huang et al. 1982 [under the name *M. ornata* ]; Jiang et al. 1995 [under the name *M. ornata* ]; Wei et al. 2013 [under the name *M. ornata* ]; Zhou et al. 2014 [under the name *M. ornata* ]; Lee et al. 2016; Chen et al. 2020; Liu et al. 2022). Several studies have shown that the dominant frequency of *M. fissipes* could be at a variation range among individuals about 1 kHz to 3 kHz (Jiang et al. 1995; Lee et al. 2016; Liu et al. 2022), with most reported populations with a mean dominant frequency of below 3 kHz (Heyer 1970 [under the name *M. ornata* ]; Huang et al. 1982; Wei et al. 2013; Chen et al. 2020; Deng et al. 2023 [estimated from the spectrogram]). However, the SW group from Hainan Island has a higher dominant frequency (mean 3.8 kHz, with several individuals' calls larger than 4 kHz) than all other reported populations. Interestingly, the SW group represents the normally diploid form according to Chen et al. (2025). That indicates no genetic distance between the SW group and the above‐mentioned population for comparison, just like that revealed by several studies (Jin et al. 2022; Yuan et al. 2016). Since the male body size of these populations was around 16 mm (Li et al. 2017), nearly reaching the threshold of so‐called “miniature frogs” of under 15 mm (Das and Haas 2010). The change in call frequency can be regarded as an indicator that the population is undergoing miniaturization, which is probably contributed to by the higher temperatures, combining the accelerated metabolic rate to rapid maturation and consequently rapid reproduction. As observed by Chen et al. (2025), the SW group also has smaller cell size and organ weight. In turn, this rapid life cycle may have contributed to the observed miniaturization of the population.

In several classic taxonomic cases, differences in karyotype and calls are the prerequisites for discovering cryptic species (e.g., Bogart and Wasserman 1972; Stöck et al. 1999). Martino and Sinsch (2002) suggested that call structure was the factor in premating isolation between diploid and tetraploid anuran species. However, the SW and NE groups of *M. fissipes* in Hainan Island did not show significant differences in pulse number and pulse rate (after corrected by temperature). Given that female frogs preferred calls from larger individuals with lower call frequencies in most anurans, it is expected there are hybrids between the tetraploid‐diploid species pair in sympatric distribution areas (Gerhardt et al. 1994). However, according to the sampling effort by Chen et al. (2025), no hybrid individuals of *M. fissipes* were obtained from Hainan Island. According to previous studies, isolation between the diploid‐tetraploid species pair was reported as behavioral adaptations such as female choice influenced by advertisement calls (Ralin 1977), character displacement (Grenat et al. 2013), calling site selection (Ptacek 1992) and aggressive behaviors (Reichert and Gerhardt 2014). Further investigations focusing on the mechanisms of acoustic isolation between this diploid‐tetraploid species pair could help understand the evolutionary mechanism of this polyploidization occurring in the island.

## Author Contributions

**Yujuan Guo:** data curation (equal), formal analysis (equal), writing – review and editing (equal). **Tianyu Qian:** data curation (equal), formal analysis (equal), methodology (lead), writing – original draft (lead). **Yulong Li:** data curation (equal), resources (equal). **Keji Guo:** resources (equal). **Wenbo Zhu:** resources (equal). **Bin Wang:** resources (equal). **Jianping Jiang:** resources (equal), supervision (equal), writing – review and editing (equal).

## Conflicts of Interest

The authors declare no conflicts of interest.

## Supporting information


**Code S1**.

**Code S2**.

Data S1.

Data S2.

Data S3.

## Data Availability

R code and raw data are provided as Supporting Information, audio files are available at figshare at: https://doi.org/10.6084/m9.figshare.28013405.
